# Host‐driven hepatic conversion of gut microbiota‐derived putrescine to spermidine mediates mannose's protective effects against hepatic steatosis in zebrafish

**DOI:** 10.1002/imt2.70101

**Published:** 2025-12-20

**Authors:** Delong Meng, Zhen Zhang, Tsegay Teame, Benjamin Earl Niemann, Rui Xia, Shichang Xu, Yajie Zhao, Yalin Yang, Chao Ran, Le Luo Guan, Zhigang Zhou

**Affiliations:** ^1^ China‐Norway Joint Lab on Fish Gut Microbiota, Institute of Feed Research, Chinese Academy of Agricultural Sciences Beijing China; ^2^ Key Laboratory for Feed Biotechnology of the Ministry of Agriculture and Rural Affairs, Institute of Feed Research, Chinese Academy of Agricultural Sciences Beijing China; ^3^ Faculty of Land and Food Systems The University of British Columbia Vancouver Canada; ^4^ Tigray Agricultural Research Institute Mekelle, Tigray Ethiopia

## Abstract

Evidence for liver metabolism of gut‐derived microbial compounds into beneficial secondary metabolites has been lacking. Here, we demonstrate that *Cetobacterium somerae* (*C. somerae*), enriched by mannose supplementation under high‐fat diet conditions, convert arginine into putrescine. The liver subsequently converts this microbial‐derived putrescine to spermidine, which functions as an effector molecule to reduce hepatic lipid accumulation. These findings uncover a novel host‐microbiota collaborative mechanism in which the arginine‐putrescine‐spermidine metabolic pathway is completed through inter‐kingdom cooperation to ameliorate hepatic steatosis.

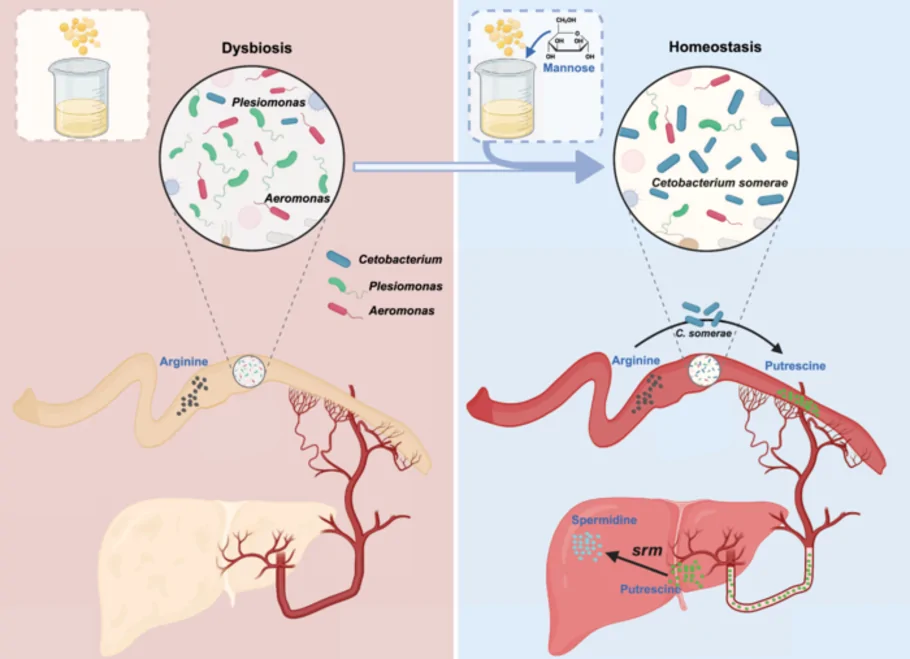

## ETHICS STATEMENT

All experiments and animal care procedures were approved by the Institute of Feed Research, Chinese Academy of Agricultural Sciences, presided over by the China Council for Animal Care (Assurance No. 2021‐AF‐FRI‐CAAS‐001).

To the Editor,

The gut microbiota interacts with its host in various ways that are crucial for maintaining the host's physiological and metabolic functions [1, 2]. The most widely known mechanisms of host–microbiota interaction are through their intrinsic cellular components or secreted compounds, such as Beta cell expansion factor A, phosphatidylethanolamine, sphingolipids, short‐chain fatty acids, and secondary bile acids [3, 4, 5, 6, 7]. These compounds have been proven to directly or indirectly influence the host's energy metabolism and immune functions [6, 7]. In addition, bacteria can utilize substrates like dietary tryptophan to produce indole derivatives that play a significant role in improving host health and fighting disease [8]. These gut microbiota metabolites can circulate in the blood to affect the functions of different organs or can be deposited in various organ systems for further processing and are considered as crucial mediators of host–microbial cross‐talk.

Recent research has revealed the gut–liver axis, involving mutual interactions between both organs and bidirectional communication through several microbial metabolites [9, 10], highlighting that bacterial metabolites derived from the gut could be affecting liver health positively or negatively. To investigate gut–liver interactions, the high‐fat diet (HFD) model is one of the most well‐established approaches [11], as a consistent HFD intake is linked to liver steatosis and gut microbiome dysbiosis [12]. This niche interaction between the gut, its microbiota, and the host liver suggests that there may be many uncharacterized metabolites produced by intestinal microbes that can be circulated to the liver to influence liver functions. Trimethylamine‐N‐oxide is one of the metabolites produced by the host liver cells from intestinal microbial product trimethylamine and has been shown to affect liver and overall health of host negatively [13]. However, no gut microbial metabolites to date have been identified that are circulated to the liver for secondary processing for host benefit. Here in this study, we identified a new gut‐liver axis mechanism using a HFD zebrafish model (Figure S1), where a key gut commensal bacterium's (*Cetobacterium somerae* (*C. somerae*)) growth and metabolism were affected by dietary mannose supplementation under HFD, and how its resulting metabolite putrescine can be further converted to spermidine by the host liver, which is proven to improve liver function via alleviation of hepatic steatosis.

## SUPPLEMENTATION OF MANNOSE ALLEVIATED HIGH‐FAT DIET‐INDUCED FATTY LIVER IN ZEBRAFISH

First, we conducted a HFD feeding experiment with mannose supplementation (Figure 1A) to determine if enriched or selected gut bacteria by it could improve liver function, as mannose was reported to alter the gut microbiota composition and alleviate fatty liver in mice [14]. After feeding trial, the weight gain and liver triacylglycerol (TAG) content of zebrafish in the HFD group were significantly increased compared to the normal‐fat diet (NFD) group, indicating the successful establishment of the nutritional fatty liver model (Figure 1B and S2A–C). When compared with the HFD group, liver TAG content was significantly lower by decreasing the expression of hepatic lipogenesis‐related genes (Figure 1B and Figure S2D,E), and the levels of serum alanine aminotransferase (ALT) and aspartate aminotransferase (AST) were significantly reduced (Figure 1C,D) in the NFD, 5 M and 10 M groups. As shown by hematoxylin and eosin (H&E) staining, the number of vacuoles representing lipid droplets and injury scores was notably reduced in 5 M and 10 M groups compared with the HFD group, while comparable with the NFD group (Figure 1E,F). The expression of liver pro‐inflammatory genes was also reduced in the 5 M and 10 M groups (Figure S2F,G). Overall, we observed that feeding a mannose‐supplemented HFD had improved intestinal health at both morphological and molecular levels in zebrafish (Figure S3A–F).

**Figure 1:**
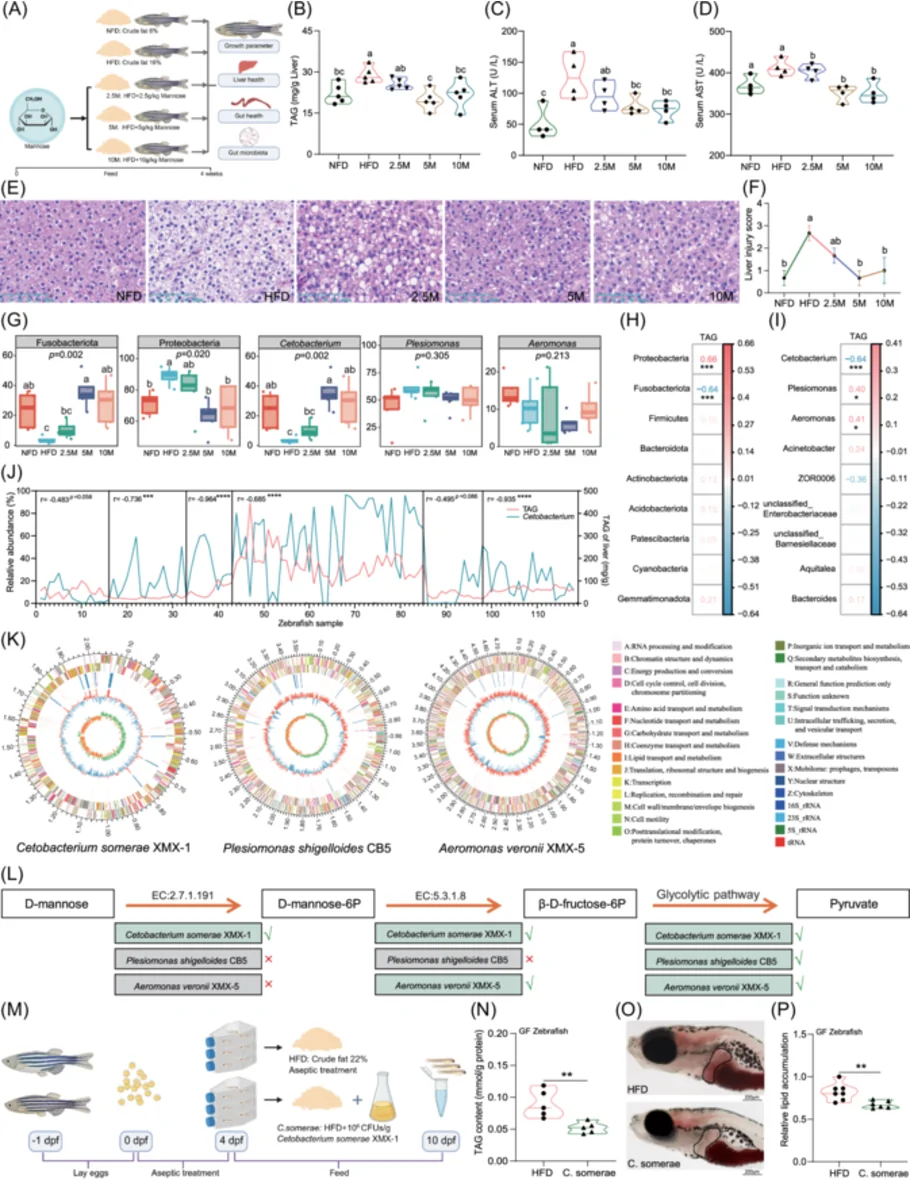
Mannose supplementation does not directly mitigate HFD‐induced liver steatosis but promotes the growth of *C. somerae*. (A) One‐month‐old zebrafish were fed normal‐fat diet (NFD), high‐fat diet (HFD), and three treatment diets supplemented with 2.5 g/kg (2.5 M), 5 g/kg (5 M), and 10 g/kg (10 M) mannose for 4 weeks, respectively. This feeding experiment was set up in five groups, where every group had five replicate tanks containing 24 zebrafish each for a total of 600 zebrafish used. (B) Triacylglycerol (TAG) content in zebrafish liver (*n* = 5). (C) Alanine aminotransferase (ALT) and (D) aspartate aminotransferase (AST) contents in zebrafish serum (*n* = 4). (E) Liver hematoxylin and eosin (H&E) sections (*n* = 3). (F) Liver injury scores (*n* = 3). (G) The relative abundance difference analysis of gut microbiota at the phylum and genus level by using Kruskal–Wallis test (*n* = 5). (H) Spearman correlation analysis between TAG content and the main phyla of gut microbiota (*n* = 25). (I) Spearman correlation analysis between TAG content and the main genera of the gut microbiota (*n* = 25). (J) The relationship between *Cetobacterium* and the liver TAG content in zebrafish by Spearman correlation analysis. (K) Chromosome circos diagram of *C. somerae* XMX‐1, *P. shigeloides* CB5, and *A. veronii* XMX‐5. (L) Annotation results of the key genes in Mannose metabolism of the three representative strains. (M) Germ‐free (GF) zebrafish larvae were directly fed a HFD supplemented with 10^6^ CFUs/g *Cetobacterium somerae* (*C. somerae*) for 7 days. Two different treatment groups (HFD and C. somerae) were used. Each group had five replicate bottles with 30 zebrafish larvae in each, for a total of 300 zebrafish larvae used. (N) TAG content of GF zebrafish larvae (*n* = 5). (O) Oil red staining of GF zebrafish larvae (*n* = 6–7). (P) Quantification of oil red staining of GF zebrafish larvae (*n* = 6–7). Data were presented as mean ± SEM or median (IQR). One‐way ANOVA was employed to compare the groups, followed by Duncan's multiple range test. Distinct letters denote significant differences at (*p* < 0.05). Significant differences were marked as *p < 0.05, **p < 0.01, ***p < 0.001. (A) and (M) were created with BioRender.com.

## MANNOSE SUPPLEMENTATION ALTERED GUT MICROBIOTA AND SELECTIVELY ENRICHED *CETOBACTERIUM*

Compared with the HFD group, the relative abundance of Proteobacteria was significantly decreased, while that of Fusobacteriota was significantly increased in the 5 M and 10 M groups (Figure 1G and Figure S4A). The relative abundance of *Cetobacterium* was significantly increased in the 5 M and 10 M groups compared to the HFD group (Figure 1G and Figure S4B). The alpha diversity indices (Chao1 and Shannon) of the gut microbiota did not differ among all groups (Figure S4C). LEfSe analysis showed that genus *Cetobacterium* was highly enriched in zebrafish gut in the 5 M group (Figure S4D). *Cetobacterium* is a genus in the phylum Fusobacteriota, represented in fish by a single species, *C. somerae*, which commonly inhabits the intestines of several fish species. Furthermore, principal coordinate analysis (PCoA) showed that the gut microbiota of HFD and 5 M groups were completely separated (Figure S4E,F), indicating that mannose supplementation significantly altered the gut microbiota composition in zebrafish. The Spearman correlation analysis showed that the relative abundance of Proteobacteria and the *Plesiomonas* and *Aeromonas* it contained were significantly positively correlated with liver TAG content, while that of Fusobacteriota and *Cetobacterium* was significantly negatively correlated (Figure 1H,I). To further verify the relationship between the abundances of *Cetobacterium, Plesiomonas*, and *Aeromonas* with liver TAG content, we collected publicly available datasets of zebrafish and *Cyprinus carpio*. Spearman correlation analysis showed that *Cetobacterium* and liver TAG content had a consistent and significant negative correlation (Figure 1J and Figure S5C), while the *Plesiomonas* and *Aeromonas* did not (Figure S5A,B and Figure S5D,E).

## GENOMIC AND GROWTH ANALYSIS OF *C. SOMERAE* REVEALED ITS ABILITY TO METABOLIZE MANNOSE

To investigate why the abundance of these different bacterial taxa in gut changed after mannose supplementation, we employed whole genome sequencing analysis of selected zebrafish gut bacteria strains, including *C. somerae* XMX‐1, *Plesiomonas shigelloides* (*P. shigelloides*) CB5, and *Aeromonas veronii* (*A. veronii*) XMX‐5. The resulting genomes verified that significant functional differences exist among the selected bacterial strains (Figure 1K and Figure S6A). First, different genomic structures were observed for the three strains (Figure 1K and Figure S6B,C). Due to the higher relative abundance of *Cetobacterium* in the gut of zebrafish fed with mannose, we then compared the metabolic pathways of monosaccharides among the three bacterial strains (Figure S6D–K). The results revealed that only *C. somerae* had the capability to metabolize mannose (Figure 1L), and further growth experiments confirmed that *C. somerae* could utilize mannose as a main carbon source for its growth (Figure S6L). In contrast, no growth was observed for *P. shigelloides* CB5 and *A. veronii* XMX‐5 when mannose was used as the main carbon source (Figure S6M,N).

## GUT *C. SOMERAE*, NOT MANNOSE, REDUCED LIVER FAT ACCUMULATION IN ZEBRAFISH

To clarify whether mannose or *C. somerae* reduced liver fat accumulation, we investigated the direct effect of mannose on reducing liver fat accumulation in germ‐free (GF) zebrafish (Figure S7A). The result verified that mannose‐supplemented diet did not reduce liver fat accumulation (Figure S7B–D), and there were no significant changes in the expression of genes related to lipid metabolism (Figure S7E,F) and inflammatory factors (Figure S7G,H). However, gut microbiota improved by mannose can reduce liver fat accumulation in zebrafish (Figure S8A–H). Then, to directly verify the effect of *C. somerae* supplementation on lowering of liver lipid accumulation, we added *C. somerae* at a concentration of 10^6^ CFUs/g to GF zebrafish larvae fed with a HFD (Figure 1M). Liver TAG content and oil red staining were significantly lowered in the *C. somerae* supplemented group compared to the HFD group (Figure 1N–P). The protective ability of *C. somerae* to the host has been confirmed in lipid metabolism and inflammation‐related genes (Figure S9A–D). Besides, the liver lipid‐lowering effects of *P. shigelloides* CB5 or *A. veronii* XMX‐5 were assessed and it was confirmed that these strains did not affect liver fat content in zebrafish (Figure S10A–D). Lastly, we found that *C. somerae* can improve liver fat accumulation and intestinal health in zebrafish on a HFD (Figure S11A–M).

## METABOLOMIC AND GENOMIC ANALYSIS REVEALED THE PRESENCE OF ARGININE TO PUTRESCINE PATHWAY IN *C. SOMERAE*

To explore what mechanism *C. somerae* could be involved with to improve overall liver health under a HFD, we combined genomic and metabolomic analysis together with an isotopic tracing approach to identify the active and causal components of *C. somerae* participating in lowering liver lipid (Figure 2A). Metabolome analysis revealed that metabolites in bacteria‐free medium and *C. somerae* supernatant were significantly separated and the intra‐group variability was low (Figure 2B and Figure S12A–C). Differential abundant metabolite analysis showed that putrescine was one of the most abundant metabolites in *C. somerae* supernatant (Figure 2C and Figure S12D–F).

**Figure 2:**
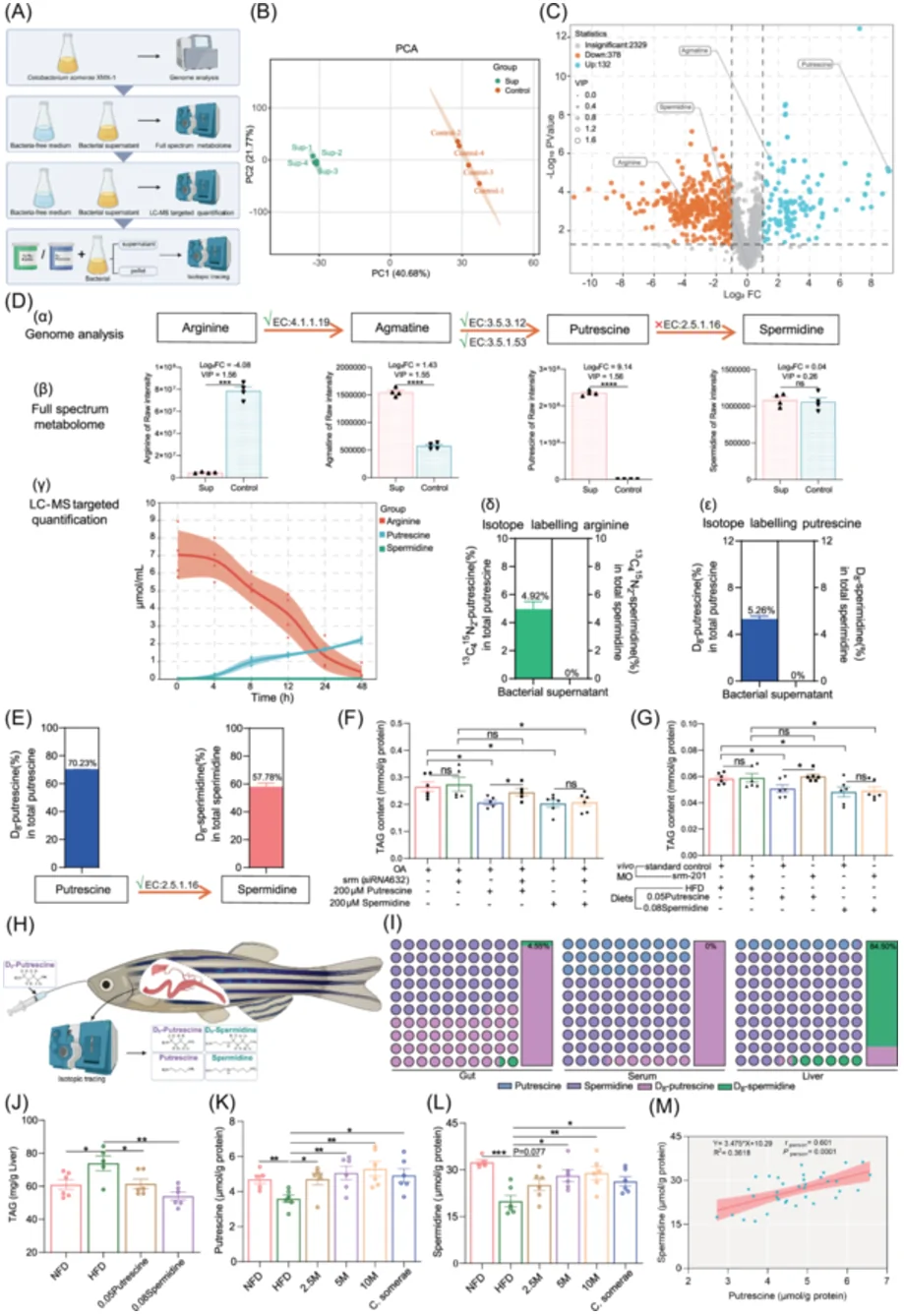
Spermidine, not putrescine, is the effector molecule that ameliorates HFD‐induced liver steatosis. (A) Diagram of methods employed to explore *C. somerae* metabolites; genomic analysis of *C. somerae* XMX‐1, full‐spectrum metabolome mining of metabolites and LC‐MS targeted quantitative analysis of key metabolites, in addition to isotope tracing. The metabolites of *C. somerae* XMX‐1 were mined using the full‐spectrum metabolome, which was divided into two groups, namely the supernatant group (Sup) and the bacteria‐free medium group (Control), with four replicates per group. (B) PCA diagram of each sample of the full‐spectrum metabolome (*n* = 4). (C) Volcano diagram of differential metabolites (*n* = 4). (D) Analysis of putrescine metabolism in *C. somerae* XMX‐1 by genome analysis on KEGG pathway (*α*), the abundance of four metabolites by full spectrum metabolome (*β*), LC‐MS targeted quantitative analysis of arginine, putrescine and spermidine in the supernatant of *C. somerae* XMX‐1 (*n* = 4) (*γ*), the contents of metabolites ^13^C_4_^15^N_2_‐putrescine, ^13^C_4_^15^N_2_‐spermidine, putrescine and spermidine in the supernatant of *C. somerae* XMX‐1 were detected after adding ^13^C_6_^15^N_4_‐arginine dihydrochloride (final concentration 680 μM) to GAM medium for 24 h (*n* = 4) (*δ*), and the contents of metabolites D_8_‐putrescine, D_8_‐spermidine, putrescine and spermidine in supernatant of *C. somerae* XMX‐1 were detected after adding D_8_‐putrescine dihydrochloride (final concentration 250 μM) to GAM medium for 24 h (*n* = 4) (*ε*). (E) Genomic analysis and isotope tracing of the ability of ZFL cells to metabolize putrescine to spermidine (*n* = 3). (F) TAG content of ZFL cells after knocking down spermidine synthase by *si*RNA 632 *n* = 5–6). (G) TAG content of zebrafish larvae after knocking down spermidine synthase by vivo MO srm‐201 (*n* = 6). (H) Zebrafish were fed with 8 μg D_8_‐putrescine (14 μg D_8_‐putrescine dihydrochloride)/fish for two consecutive days, and the content of the metabolites D_8_‐putrescine, D_8_‐spermidine, putrescine, and spermidine was detected in the gut, serum and liver of zebrafish after the last feeding for 2 h (*n* = 4). (I) The content of putrescine (0.065 ± 0.023 μmol/g protein), spermidine (16.659 ± 0.767 μmol/g protein), D_8_‐putrescine (11.889 ± 3.568 μmol/g protein, residual after feeding) and D_8_‐spermidine(0.473 ± 0.046 μmol/g protein) in the gut, the content of putrescine (37.526 ± 4.324 μmol/L), spermidine (97.160 ± 11.449 μmol/L), D_8_‐putrescine (9.008 ± 1.365 μmol/L) and D_8_‐spermidine(no test) in the serum, the content of putrescine (3.632 ± 0.336 μmol/g protein), spermidine (31.224 ± 4.158 μmol/g protein), D_8_‐putrescine(0.376 ± 0.117 μmol/g protein) and D_8_‐spermidine (2.072 ± 0.656 μmol/g protein) in the liver. (J) One‐month old zebrafish were fed with diets supplemented with 0.5 g/kg putrescine (0.05putrescine) and 0.8 g/kg spermidine (0.08spermidine) (with equimolar amounts of putrescine and spermidine) for four weeks. TAG content in zebrafish liver (*n* = 5–6). (K) The content of putrescine in liver of Zebrafish fed with mannose and *C. somerae* (*n* = 5–6). (L) The content of spermidine in liver of Zebrafish fed with mannose and *C. somerae* (*n* = 5–6). (M) Linear regression analysis of the liver content of putrescine and spermidine in Zebrafish fed with mannose and *C. somerae* (*n* = 35). Data were presented as mean ± SEM. The student's *t*‐test was employed to compare between two groups. Significant differences were marked as *p < 0.05, **p < 0.01, ***p < 0.001, ****p < 0.0001, ns = not significant. (A), (H) were created with BioRender.com.

We then annotated the main enzymes in the putrescine metabolic pathway based on the genome sequences of *C. somerae*, and found that *C. somerae* possesses genes encoding arginine decarboxylase (an enzyme that metabolizes arginine to agmatine) and genes (agmatine deiminase and *N*‐carbamoylputrescine amidase) to produce putrescine from agmatine (Figure 2Dα) as reported in other bacteria [15]. In addition, the full‐spectrum metabolome analysis of *C. somerae* showed that this bacterium could utilize arginine to produce agmatine and further produce putrescine (Figure 2Dβ). Further targeted LC‐MS analysis showed that arginine was fully utilized by *C. somerae* at 48 h, and the content of putrescine reached 2.22 μmol/mL (Figure 2Dγ). To confirm this metabolic pathway was present in *C. somerae*, exogenous isotope‐labeled ^13^C_6_^15^N_4_‐arginine was added to the culture medium, and the results showed that ^13^C_4_^15^N_2_‐putrescine was detected in the supernatant and bacterial pellet of *C. somerae* (Figure 2Dδ and Figure S12G). Finally, D_8_‐putrescine was supplemented to the culture medium to verify whether *C. somerae* can further metabolize putrescine to produce spermidine. The results showed that D_8_‐putrescine was detected in the supernatant and bacteria pellet of *C. somerae*, while no labeled spermidine was present (Figure 2Dε and Figure S12H). The joint analyses revealed that *C. somerae* can produce putrescine through the arginine metabolic pathway, which may be the potential metabolite exerting an effect on lowering liver lipid.

## PUTRESCINE HAD NO DIRECT EFFECT ON LIVER FUNCTION, BUT ITS DOWNSTREAM HOST METABOLITE SPERMIDINE ALLEVIATED FATTY LIVER PATHOLOGY

Spermidine [16], a downstream metabolite of putrescine, has been shown to alleviate liver steatosis [17], but the spermidine synthase (*srm*) gene was not found in *C. somerae*'s genome, and it was not detected in the bacterial supernatant. Furthermore, no ^13^C_4_^15^N_2_‐spermidine and D_8_‐spermidine were detected in any components of the *C. somerae* cultures (Figure 2Dα‐ε). First, we examined the zebrafish genome and found that it does possess a spermidine synthase (EC:2.5.1.16) gene that can metabolize putrescine into spermidine. Besides, an isotope tracing experiment using zebrafish liver (ZFL) cells model showed that 57.78% D_8_‐spermidine in total spermidine were detected in ZFL cells (Figure 2E and Figure S13A), indicating that the ZFL cells could metabolize D_8_‐putrescine to D_8_‐spermidine. We then verified if putrescine and spermidine could affect liver lipid metabolism in a ZFL HFD model (Figure S13B). Compared with the control, the level of intracellular TAG content was significantly decreased in the putrescine and spermidine‐treated ZFL cells (Figure S13C–H). To validate the effect of spermidine on liver function, we knocked down spermidine synthase in the ZFL cells and zebrafish larvae. After silencing spermidine synthase, the lipid‐lowering effect disappeared in the putrescine‐treated group, while it remained in the spermidine‐treated group (Figure 2F,G and Figures S13I–L, S14A–C).

## DISCOVERY OF PUTRESCINE‐SPERMIDINE CROSS‐TALK IN THE HFD ZEBRAFISH MODEL

To verify if gut microbial putrescine could be translocated to the liver to be used as a precursor to synthesize spermidine (Figure 2H). The in vivo isotope tracing experiment showed that D_8_‐putrescine in the gut could be transported to the liver through the blood, and the liver could metabolize 84.50% of D_8_‐putrescine into D_8_‐spermidine, and the content of spermidine was much higher than putrescine in the liver (Figure 2I). The liver lipid‐lowering effects of putrescine and spermidine were further verified using HFD zebrafish model (Figure S15A). Compared with the HFD group, the liver TAG content in both the putrescine and spermidine treated groups was significantly reduced (Figure 2J and Figure S15B–F). The levels of putrescine and spermidine in the liver of both the putrescine‐ and spermidine‐treated groups returned to the normal (Figure S15G–N). Furthermore, this was also confirmed in the mannose and *C. somerae* supplemented groups (Figure 2K–M), indicating that the liver is the main site for conversion of putrescine to spermidine.

In conclusion, this study identified a novel mechanism involving an arginine‐putrescine‐spermidine cascade metabolic pathway that improves liver steatosis through the collective interaction between the gut *C. somerae* and the host liver. Our findings on the novel microbe–host metabolic interplay in the gut‐liver axis to produce a useful metabolite, spermidine, have led to a new concept of host–microbe cross‐talk in the gut‐liver axis.

## AUTHOR CONTRIBUTIONS

**Delong Meng**: Writing—original draft; writing—review and editing; software; formal analysis; validation; investigation; data curation; visualization; methodology. **Zhen Zhang**: Writing—original draft; writing—review and editing; project administration; data curation; supervision; funding acquisition. **Tsegay Teame**: Writing—review and editing. **Benjamin Earl Niemann**: Writing—review and editing. **Rui Xia**: Software; validation. **Shichang Xu**: Software; validation. **Yajie Zhao**: Software; validation. **Yalin Yang**: Supervision; data curation. **Chao Ran**: Supervision; data curation. **Le Luo Guan**: Writing—review and editing; supervision; software. **Zhigang Zhou**: Supervision; funding acquisition; conceptualization; project administration; resources. All authors have read the final manuscript and approved it for publication.

## CONFLICT OF INTEREST STATEMENT

The authors declare no conflicts of interest.

## Supporting information


**Figure S1.** The series of experiments conducted in this study.
**Figure S2.** The effects of diet supplementation of mannose on growth performance and liver health of zebrafish.
**Figure S3.** The effects of diet supplementation of mannose on the gut health of zebrafish.
**Figure S4.** The effects of diets supplementation of different levels of mannose on gut microbiota of zebrafish.
**Figure S5.** Meta analysis fish gut microbiota.
**Figure S6.** Genomic analysis representative strains of fish gut.
**Figure S7.** The effect of mannose supplementation on zebrafish liver steatosis.
**Figure S8.** The effect of supplementation of mannose induced gut microbiota on zebrafish liver steatosis.
**Figure S9.** The effect of *C. somerae* supplementation on zebrafish liver steatosis.
**Figure S10.** The effect of supplementation of *P. Shigeloides* and *A. veronii* on zebrafish liver steatosis.
**Figure S11.** The effect of *C. somerae* supplementation on zebrafish liver steatosis.
**Figure S12.** Metabolite analysis of *C. somerae* XMX‐1.
**Figure S13.** The metabolism of putrescine to spermidine exerts the effect of lowering liver fat in ZFL cells.
**Figure S14.** The metabolism of putrescine to spermidine exerts the effect of lowering liver fat in zebrafish larvae.
**Figure S15.** The mechanism of putrescine and spermidine on reducing liver fat in zebrafish.
**Figure S16.** The polyamine metabolism in zebrafish.
**Table S1.** Ingredients (g/100 g diet) and chemical compositions (%) of zebrafish diets.
**Table S2.** Ingredients (g/100 g diet) and chemical compositions (%) of zebrafish larvae diets.
**Table S3.** A real‐time PCR amplification system.
**Table S4.** A real‐time PCR amplification program.
**Table S5.** List of primer sequences used for qPCR.

## Data Availability

The data that support the findings of this study are available from the corresponding author upon reasonable request. Microbiota sequencing and bacterial whole genome sequence data for this study were made available from the National Center for Biotechnology Information (NCBI) under BioProject accession numbers PRJNA1203443 (https://www.ncbi.nlm.nih.gov/bioproject/PRJNA1203443), PRJNA1205406 (https://www.ncbi.nlm.nih.gov/bioproject/PRJNA1205406), PRJNA1205539 (https://www.ncbi.nlm.nih.gov/bioproject/?term=PRJNA1205539), and PRJNA1205650 (https://www.ncbi.nlm.nih.gov/bioproject/?term=PRJNA1205650), respectively, and are publicly available as of the date of publication. Similarly, the original microbiota sequence data for meta‐analysis were saved under BioProject accession numbers PRJNA1222012 (https://www.ncbi.nlm.nih.gov/bioproject/PRJNA1222012) and PRJNA1222015 (https://www.ncbi.nlm.nih.gov/bioproject/PRJNA1222015). Supplementary materials (figures, tables, graphical abstract, slides, videos, Chinese translated version, and update materials) may be found in the online DOI or iMeta Science http://www.imeta.science/.
