# Supplementary material for: Host‐driven hepatic conversion of gut microbiota‐derived putrescine to spermidine mediates mannose's protective effects against hepatic steatosis in zebrafish
Source: Imeta. 2025 Dec 20;5(1):e70101. doi: 10.1002/imt2.70101 (PMC13726591; doi:10.1002/imt2.70101)
Supplement: Supplementary file 1 [file IMT2-5-e70101-s001.docx]

Supporting information to

**Host-driven hepatic conversion of gut microbiota-derived putrescine to spermidine mediates mannose’s protective effects against hepatic steatosis in zebrafish**

**Running title:** Gut-liver interplay to ameliorate hepatic steatosis

Delong Meng^1,$^, Zhen Zhang^2,3,$,*^, Tsegay Teame^1,4^, Benjamin Earl Niemann^3^, Rui Xia^1^, Shichang Xu^1^, Yajie Zhao^1^, Yalin Yang^2^, Chao Ran^2^, Le Luo Guan^3,*^, Zhigang Zhou^1,*^

^1^China-Norway Joint Lab on Fish Gut Microbiota, Institute of Feed Research, Chinese Academy of Agricultural Sciences, Beijing, 100081, China

^2^Key Laboratory for Feed Biotechnology of the Ministry of Agriculture and Rural Affairs, Institute of Feed Research, Chinese Academy of Agricultural Sciences, Beijing, 100081, China

^3^Faculty of Land and Food Systems, The University of British Columbia, Vancouver, Canada

^4^Tigray Agricultural Research Institute, Mekelle, Tigray, Ethiopia

^$^These authors contributed equally to this work.

*Corresponding author. Email: zhouzhigang03@caas.cn; leluo.guan@ubc.ca; zhangzhen@caas.cn

**SUPPLEMENTAL RESULTS**

**Diet supplementation of mannose improved intestinal health in zebrafish**

When the gut functions were investigated, the intestinal morphology and villus height were improved (Figure S3A, B), while serum lipopolysaccharides (LPS), a marker of intestinal permeability, was notably reduced (Figure S3C) in 5M and 10M mannose supplemented groups compared to HFD group. In addition, the expression of *occludin b* was notably higher in 5M and 10M groups (Figure S3D). Furthermore, the expression of pro-inflammatory genes in the gut of zebrafish fed with mannose-supplemented diets were significantly lower (Figure S3E), while the expression anti-inflammatory genes did not differ among all groups (Figure S3F). Overall, we observed that feeding a mannose supplemented HFD had improved intestinal health at both morphological and molecular levels in zebrafish.

**Genomic analysis of three native bacteria in the fish intestine**

Specifically, different genomic structures were observed for the three stains: *C. somerae* XMX-1’s genome consisted of 1 chromosome and 6 plasmids (Figure 1K and S6B); *P.* *shigelloides* CB5’s consisted of 1 chromosome and 1 plasmid (Figure 1K and S6B); and *A. veronii* XMX-5’s consisted of 1 chromosome only (Figure 1K). Although the Kyoto Encyclopedia of Genes and Genomics (KEGG), Clusters of Orthologous Genes (COG) and Carbohydrate-active enzymes (CAZymes) analysis showed similar major functions in carbohydrate metabolism and transport among the three strains (Figure S6E-K), comparative genomic analysis revealed that *C. somerae* XMX-1, *P. shigelloides* and *A. veronii* XMX-5 CB5 possessed 1239, 938 and 1635 unique genes, respectively, and 853 genes were shared among them, indicating the existence of functional differences among the bacteria strains (Figure S6D).

**Gut microbiota** **improved by mannose can reduce liver fat accumulation in zebrafish**

To verify whether the specific mannose-enriched gut microbiota lowered liver fat accumulation, we transferred the gut microbiota from zebrafish fed with a HFD (HFD-GM) or a HFD supplemented with mannose (HFD-MO-GM) to GF zebrafish larvae (Figure S8A). The results showed that liver TAG content was significantly reduced in the HFD-MO-GM group compared to the HFD-GM and GF groups (Figure S8B). Oil red staining exhibited less abundant lipid droplet accumulation in the liver tissue of HFD-MO-GM versus GF and HFD-GM groups, and this was also verified by higher mean gray values (Figure S8C, D). In addition, the expression of lipogenesis genes (*pparγ*, *fas*, *dgat2*) and lipolysis genes (*pparα*, *cpt1*) were significantly lower in the liver of the HFD-MO-GM group compared to the HFD-GM and GF groups (Figure S8E, F). Furthermore, the expression of pro-inflammatory genes (*nf-κb p65*, *il-6*) in the HFD-MO-GM group were significantly downregulated, while that of the anti-inflammatory gene (*i1-10*) was notably upregulated (Figure S8G, H).

**Diet supplementation of *C. somerae* improved liver and gut health in zebrafish**

Lastly, we compared the same parameters in adult zebrafish fed with HFD and HFD supplemented with 10^6^ CFUs/g *C. somerae* (Figure S11A). Compared with the HFD group, the *C. somerae* supplemented group of zebrafish liver showed notably lower level of TAG content (Figure S11B). H&E staining of the liver showed a significantly lower number of vacuoles representing lipid droplets, and the liver injury scores were reduced considerably (Figure S11C, D) in the *C. somerae* supplemented group. The expression of lipogenesis genes (*c/ebpα*, *acc*, *fas*) and lipolysis gene (*pparα*) was significantly downregulated in the liver of the zebrafish supplemented with *C. somerae* compared to the HFD group (Figure S11E, F). In addition, the expression of liver pro-inflammatory gene (*il-1β*) in the *C. somerae* supplemented group was markedly downregulated, whereas the expression of the anti-inflammatory gene (*i1-10*) was notably upregulated in zebrafish supplemented with *C. somerae* (Figure S11G, H). Moreover, serum ALT and AST (Figure S11I, J) as well as LPS (Figure S11K) concentrations were significantly reduced in zebrafish supplemented with *C. somerae* compared to the HFD group. Furthermore, H&E staining of intestinal tissues showed that *C. somerae* supplementation increased villi height and led to closer physical arrangement of the villi (Figure S11L), as well as reduced gut injury scores (Figure S11M). The above data demonstrate that mannose supplementation alone has no direct effect on lowering of liver lipids and improvement of liver health, but has the ability to promote the abundance of intestinal *C. somerae*, which can reduce liver fat accumulation and alleviate inflammation through an undefined mechanism.

**Metabolomic analysis *C. somerae***

Full-spectrum metabolome analysis revealed that the metabolite composition in the culture supernatant of *C. somerae* were with high quality and reproducibility to identify the active and causal components participating in lowering liver lipid (Figure S12A, B). Principal component analysis (PCA) and hierarchical cluster analysis showed that the metabolites in bacteria-free medium (Control) and *C. somerae* supernatant (Sup) were significantly separated and the intra-group variability was low (Figures 2B and S12C). In total, 3043 and 3038 metabolites were detected in the Control and Sup groups respectively. Among them, 2975 metabolites were shared by the two groups, and 63 metabolites were unique to the supernatant (Figure S12D). Furthermore, clustering heat map of differential metabolites analysis showed that there were many metabolites between the two groups mainly involved in multiple metabolic pathways, including amino acid and its metabolites, organic acid and its derivatives, alcohol and amines, etc., (Figure S12E).

**Putrescine had no direct effect on liver function, but its downstream host metabolite spermidine alleviated fatty liver pathology**

The expression of lipogenesis genes (*srebp-1c*, *c/ebpα*, *pparγ*, *acc*, *fas* and *dgat2*) were markedly reduced in ZFL cells treated with putrescine and spermidine (Figure S13D), and the expression of lipolysis genes (*pparα*, *cpt1* and *acox3*) were also significantly reduced (Figure S13E). Furthermore, compared with the Control group, the expression of pro-inflammatory genes (*nf-κb p65*, *il-1β* and *il-6*) was significantly reduced in the putrescine and spermidine treated ZFL cells, while the expression of anti-inflammatory gene (*il-10*) was significantly increased in the spermidine treated ZFL cells (Figure S13F, G). In addition, after supplementing putrescine and spermidine, the expression of the *srm* gene in ZFL cells was significantly upregulated (Figure S13H). To validate the effect of spermidine on liver function (Figure S13I), we knocked down spermidine synthase in the ZFL cells model using *si*RNA 632. After silencing spermidine synthase, the hepatic lipid-lowering effect of the putrescine was disappeared (Figure 2F), while in the spermidine supplemented group, the process was not affected. The same results were observed with two other spermidine synthase inhibitors, 2-mercaptoethylamine and cyclohexylamine (Figure S13J-L).

Next, we knocked down the spermidine synthase gene in zebrafish larvae using *vivo* morpholino oligonucleotide srm-201 (Figure S14A). The results showed that the TAG content was significantly decreased in the putrescine and spermidine treated groups, compared to the HFD group. After silencing spermidine synthase, the lipid-lowering effect disappeared in the putrescine treated group, while it remained in the spermidine treated group (Figure 2G). Liver oil red staining also verified that the lipid droplet accumulation increased and the average gray value was lower in the putrescine treated group compared to before silencing spermidine synthase (Figure S14B, C). The spermidine treated group was not affected before and after silencing spermidine synthase (Figure S14B, C). These results demonstrated that putrescine could not lower liver lipids on its own but the host do this by metabolizing putrescine into spermidine, which is an effector molecule in liver lipid lowering processes.

**Discovery of putrescine-spermidine cross talk in the HFD zebrafish model**

The expression of lipogenesis genes (*srebp-1c* and *fas*) in the liver of putrescine and spermidine treated groups were significantly downregulated (Figure S15C). In addition, the expression of lipogenesis genes (*c/ebpα*, *pparγ*, *acc*) was notably downregulated in the spermidine treated group (Figure S15C), whereas no significant change was observed in the expression of lipolysis genes in both groups (Figure S15D). Besides, the expression of pro-inflammatory genes (*nf-κb p65* and *tnf-α*) were significantly downregulated in the putrescine and spermidine treated groups compared to the HFD group (Figure S15E, F). The levels of putrescine and spermidine in the livers of both the putrescine- and spermidine-treated groups returned to the normal, comparable to those in the NFD group, and were significantly higher than those in the HFD group (Figure S15G, H). A significant positive correlation was observed between the contents of putrescine and spermidine in the liver (Figure S15I). Compared with the HFD group, the expression of the spermidine synthase protein was significantly upregulated in the putrescine and spermidine treated groups (Figure S15L, M). Similarly, the expression of polyamine metabolism pathway related genes (*arg2, odc1, srm* and *sms*) was significantly upregulated in the putrescine and spermidine treated groups compared to the HFD group (Figure S15N).

In addition, putrescine was much higher than spermidine in the feces of healthy adults, and the main polyamine produced by the gut microbiota was putrescine [1], which was consistent in the detection of polyamine metabolites in the gut contents of zebrafish (Figure S16A). Our study confirmed that over 80% of the D_8_-putrescine from zebrafish gut contents was converted to the D_8_-spermidine in the liver (Figure 2I), suggesting that putrescine, a main metabolite of the gut microbiota, was further metabolized to spermidine by the host liver. Moreover, we found that the highest concentration polyamine metabolite in the zebrafish liver was spermidine (compared to putrescine and spermine) (Figure S15J, K). Similarly, a study confirmed the highest level of polyamine metabolite in human liver was spermidine [2]. The expression of spermidine synthase may be more closely related to liver injury, that is, spermidine, not putrescine is the main active substance that alleviates liver fat accumulation. Previous studies have shown that spermidine can be produced by the gut microbial species independently or collaboratively to regulate host liver health [3, 4]. Compared to these findings, our results confirmed the role of gut microbiota and host collaboration in the production of spermidine (Figure S16B, C).

**MATERIALS AND METHODS**

**Zebrafish (*Danio rerio*) studies**

All experiments and animal care procedures were approved by the Institute of Feed Research of Chinese Academy of Agricultural Sciences, presided over by the China Council for Animal Care (Assurance No. 2021-AF-FRI-CAAS-001). One-month-old AB strain zebrafish with similar body weight were used in this experiment. Zebrafish were randomly allocated to 3L tanks (24 fish per tank). Specifically, the experiment in Figure 1A consisted of 5 groups with 5 replicate tanks in each group, and a total of 600 zebrafish were used. The experiment in Figure S11A consisted of 2 groups with 5 replicate tanks in each group, and 240 zebrafish were used in total. The experiment in Figure S15A consisted of 4 groups with 5 replicate tanks in each group, and a total of 480 zebrafish were used. The experiment in Figure 2K-M consisted of 6 groups with 5 replicate tanks in each group, and a total of 720 zebrafish were used. The feed formula is shown in Table S1 (different formulations for each treatment group, see figure legend for details). Zebrafish were fed twice a day, at 9:00 and 16:00, for 4 weeks. The feeding regimen consisted of 4% of their initial body weight in week 1, 6% in weeks 2 and 3, and 8% in week 4. During the experiments, the circulating water parameters (tanks on static) were set as follows: temperature of approximately 28℃, flow rate was 120 L/h, pH about 7.2, dissolved oxygen levels above 6.0 mg/L, nitrite content levels under 0.05 mg/L, ammonia nitrogen levels below 0.5 mg/L.

Zebrafish larvae were produced by breeding of AB strain zebrafish in our laboratory. The eggs were kept in the incubation tank for 4 dpf (days post fertilization) until most of them hatched, then 30 zebrafish larvae were transferred to each culture bottle with 30 mL filtered water. Specifically, the experiment in Figure S7A consisted of 2 groups with 5 replicate bottles in each group, and for a total of 300 zebrafish larvae were used. The experiments in Figure S8A and S10A consisted of 3 groups with 5 replicate bottles in each group, and a total of 450 zebrafish larvae were used. The experiment in Figure 1M consisted of 2 groups with 5 replicate bottles in each group, and a total of 300 zebrafish larvae were used. The experiment in Figure S14A consisted of 6 groups with 6 replicate bottles in each group, and a total of 1080 zebrafish larvae were used. The high-fat model of zebrafish larvae was created by feeding at 5 dpf twice a day and changing the water at 9:00 and 16:00. The feed formula is shown in Table S2 (different formulations for each treatment group, see figure legend for details).

**Generation of Germ-free zebrafish**

Germ-free (GF) zebrafish were generated following the established protocol described previously [5, 6].

**Zebrafish liver cells culture**

Methods of culture and passage of zebrafish liver (ZFL) cells (ATCC number CRL-2643) were prepared as described previously [7].

**Bacterial culture**

Three bacterial species including *Cetobacterium somerae* XMX-1 (CGMCC no.18908), *Plesiomonas* *shigelloides* CB5 (CLAM629) and *Aeromonas veronii* XMX-5 (CLAM126), were isolated from the intestine of zebrafish in our laboratory and stored at the General Microbiology Center of China Microbial Species Preservation Management Committee and National Collection of Livestock and Aquatic Microbes (Beijing), respectively. The culture of *C. somerae* XMX-1 was incubated anaerobically at 28℃ for 24 h using Gifu Anaerobic Medium (GAM) in an anaerobic incubator (Electrotek, England). The culture of *P.* *shigelloides* CB5 and *A. veronii* XMX-5 were carried out in an oscillating incubator (Zhichu, China) at 28℃ in Luria-Bertani Medium (LB) for 24 h.

**Growth index detection**

Each tank of zebrafish was weighed and counted to obtain initial body weight (IBW, g), final body weight (FBW, g), number of fish at the start/end of the experiment (denoted as N_s_ and N_e_, respectively). Similarly, the food intake (g) was recorded in each tank. The survival rate (SR, %), weight gain (WG, %) and feed conversion ratio (FCR) were calculated using the following formulas:

$$Survival rate (\%) =\frac{N_{e}}{N_{s}}\times100$$

$$\text{Weight gain (\%)}\text{ }=\frac{\mathrm{FBW}-\mathrm{IBW}}{\mathrm{FBW}}\times100$$

$$Feed conversion ratio (FCR)=\frac{Food intake}{\mathrm{FBW}-\mathrm{IBW}}$$

**Triacylglycerol assay**

Triacylglycerol (TAG) was extracted from the tissue samples and quantified with reference to previous methods [8]. The concentration of liver TAG was expressed as mg/g liver weight. Free glycerol reagent (Sigma) and triglyceride reagent (Sigma) were configured as liquid A in a 4:1 ratio, and free glycerol reagent and double distilled water were configured as liquid B also at 4:1. The glycerol standard solution (Sigma) was diluted in 8 concentrations at 2x ratio. Then standard and sample were added to 96 well enzyme labeled plates (10 μL per well), and each sample was added to 2 wells, 1 well with 200 μL liquid A and 1 well with 200 μL liquid B. After sample addition, it was incubated at 37℃ for 10 min, and absorbance was detected at 540 nm. The TAG content in the sample was obtained according to the standard curve. The weight of the liver was weighed in advance.

In addition, the TAG content of ZFL cells and zebrafish larvae were detected by enzymatic assay kit (Applygen, Beijing) and the content of protein was quantified by the Bicinchoninic acid (BCA) method (Beyotime). To avoid the head TAG interference of zebrafish larvae, the TAG content was detected after the larvae head was removed. The TAG content of ZFL cells and zebrafish larvae was expressed as mmol/g protein.

**Detection of serum parameters**

To analyze one serum parameter, blood samples of 6 zebrafish were centrifuged to obtain one sample, with 4 replicates from each group (total of 24 zebrafish). The tail of the zebrafish was removed and the caudal peduncle was put into a 500 μL centrifuge tube, which was then put into a 1.5 mL centrifuge tube and centrifuged at 2,000 rpm for 3 min to collect blood. After being placed on ice for 10 min, the upper serum was collected by centrifugation at 4,000 rpm for 10 min and serum parameters were detected. The contents of alanine aminotransferase (ALT), aspartic aminotransferase (AST) and lipopolysaccharides (LPS) in serum were determined according to the instructions of the ALT and AST activity detection kit (Jiancheng, Nanjing) and ToxinSensor™ Chromogenic LAL Endotoxin Assay Kit (Genscript).

**Liver and intestine histology analysis and liver lipid accumulation assessment using Oil Red O staining**

Liver and intestine of zebrafish were cleaned with sterile phosphate buffered saline (PBS) and fixed with 4% paraformaldehyde, then embedded in paraffin, sliced, and stained with hematoxylin and eosin to be observed under a microscope (Leica DMIL-LED, Germany). The grades given are between 0 and 3 (0 = Normal, 1 = Slight disruption, 2 = Moderate disruption and 3 = Severe disruption) based on the structure of the liver and intestine.

After fixing zebrafish larvae and adult zebrafish liver with 4% paraformaldehyde, they were washed twice in PBS and stained with 0.5% Oil red O at room temperature, away from light. The specific method can be referred to in previous studies [8]. Images were taken under light microscope (Leica DMIL-LED, Germany), and Oil red O staining was quantitatively analyzed using ImageJ software.

**Quantitative real-time PCR analysis**

Total RNA of zebrafish liver and intestine, zebrafish larvae and ZFL cells was extracted using TRIzol (CWBIO), and 2μg of RNA was used for reverse transcription into cDNA (FastKing gDNA Dispelling RT SuperMix, Tiangen). Real-time PCR was performed on a Light Cycler 480 (Roche) using cDNA as template with 2× Fastfire Plus PreMix (SYBR Green, Tiangen). The amplification system and program are shown in Table S3 and Table S4, respectively. Primer sequences are shown in Table S5 (synthesized by Shanghai Shenggong Biotechnology Company). *Ribosomal protein s11* gene (*rps11*) was used as the internal reference gene, and the data was analyzed using the 2^-ΔΔCT^ method.

**Gut microbiota sequencing**

Zebrafish gut contents were collected under aseptic conditions within 4 to 6 h of feeding, and total DNA was extracted. Primers 338F (5'-ACTCCTACGGGAGGCAGCA-3') and 806R (5'-GGACTACHVGGGTWTCTAAT-3') were used to amplify the 16S V3-V4 region. Samples were sequenced (250 bp paired end, amplification region length threshold 350bp-490bp) and denoised on an Illumina Novaseq 6000 platform (Illumina), and assembled into Amplicon Sequence Variants (ASVs). All the data were analyzed using the online platform of BMKCloud (www.biocloud.net) from Beijing Biomarker Technologies Co., Ltd. The correlation between gut microbiota and TAG content was analyzed using Spearman correlation. Microbiota sequencing data can be obtained from the National Center for Biotechnology Information (NCBI), BioProject accession number PRJNA1203443.

**Whole genome sequencing and de novo assembly of bacteria**

Bacterial DNA extraction kit (Qiagen) was used to extract DNA according to the manufacturer’s protocol. Purified genomic DNA was quantified and high-quality DNA was used to do further research. The Oxford Nanopore Technology sequencer PromethION was used to conduct single-molecule DNA sequencing and obtain original long reads for assembly. The original sequencing data was assembled using SOAPdenovo and Unicycler. All analyses were performed using the online platform of Majorbio Cloud Platform (http://cloud.majorbio.com) from Shanghai Majorbio Bio-pharm Technology Co., Ltd. Bacterial whole genome sequence data can be obtained from NCBI with BioProject accession numbers PRJNA1205406, PRJNA1205539 and PRJNA1205650, respectively.

**Bacteria utilize mannose as a substrate**

We customized a GAM (Coolaber, Beijing) with no obvious carbon source and added an additional 8 g/L mannose to the medium. OD_600_ was detected at 4, 8, 12, 24 and 36 h to determine the growth of *C. somerae* XMX-1, *P.* *shigelloides* CB5 and *A. veronii* XMX-5 in an anaerobic incubator at 28℃.

**Detection of metabolites of *C. somerae***

The culture of *C. somerae* XMX-1 was incubated anaerobically at 28℃ for 24 h using GAM. Separation of bacterial pellet and supernatant was performed by centrifugation at 12,000 rpm for 3 min. The obtained bacterial pellet was washed in ultra-pure water 3 times. The creation of the bacterial pellet lysate was accomplished by incubating on ice for 10 min, followed by ultrasound (300 W) for 10 min. Bacterial supernatant was obtained from *C. somerae* XMX-1 culture, and GAM medium was used as the control. Hydrophilic and hydrophobic substances were extracted, and full spectrum metabolome was determined by LC-MS/MS. The test was accomplished by Wuhan Metware Biotechnology Co., Ltd.

**Gut microbiota transfer to germ-free zebrafish**

Transfer of adult zebrafish gut microbiota to GF zebrafish was performed following the previously established method [9]. In the single bacterium transfer experiment, the cultured *C. somerae* XMX-1 was centrifuged at 12,000 rpm for 10 min to harvest bacterial cells. The collected bacterial cells were washed three times with distilled PBS. At a final concentration of 10^6^ CFUs/mL, GF zebrafish were colonized at 4 dpf. Before providing the diets for GF zebrafish, 20 kGy γ-ray irradiation was applied to sterilize the feed.

**LC-MS targeted quantification of Arginine, Putrescine and Spermidine**

A 1.5 mL saturated sodium bicarbonate solution and 1mL Dan sulfonyl chloride solution (10mg/mL, soluble in acetone) were added to 50 μL of sample. The solution was then put on a shaker at 40℃ for 1 h away from direct light. After this, 100 μL of ammonia was added to stop the reaction. A 1 mL acetonitrile was added to the reaction solution, which was then centrifuged at 12,000 rpm for 15 min and filtered using a 0.22 μm membrane to obtain the supernatant. Arginine (Yuanye, shanghai), Putrescine (Anpel, shanghai), Spermidine (Anpel, shanghai), Spermine (Anpel, shanghai) and D_8_-Putrescine dihydrochloride (GlpBio) were used to construct the standard curve. The supernatant was then subjected to LC-MS analysis to quantify the levels of Arginine, Putrescine, Spermidine, Spermine, D_8_-Putrescine and D_8_-Spermidine in the supernatant.

Liquid chromatography separation was performed on a Symmetry C18 column (250 mm × 4.6 mm i.d.; 5 μm; Waters, Ireland) using an Agilent 1260 Series system (Agilent, USA). The total flow rate was 1.0 mL/min; mobile phase A consisted of water with 0.1% formic acid and mobile phase B consisted of acetonitrile. The total elution program was 33 min. The gradient commenced at 50% mobile phase B, increased to 100% B over 20 min, held for 7 min and returned to 50% B in 0.1 min, and re-equilibrates for 5.9 min before the next injection. The column temperature and volume of injection were set at 35°C and 10 μL, respectively.

The Agilent Accurate-Mass-Q-TOF MS 6520 system equipped with an Electrospray ionization (ESI) source was used for mass spectra in the positive ionization mode. The parameters were as follows: a fragmentor voltage of 130 V was used; a capillary voltage of 3500 V; a nitrogen flow rate of 11 L/min and nebulizer pressure of 45 psi. In the scan range of m/z 80-2000, full-scan spectra were obtained at a rate of 1.03 spectra/s. Data were qualitatively and quantitatively analyzed using MassHunter Workstation Software (Agilent Technologies, Inc. 2012).

**Zebrafish liver cells gene knockdown**

The ZFL cells lipid accumulation model was constructed by adding 100 μmol/L oleic acid (Sigma-Aldrich) to the culture medium. For ZFL cells, spermidine synthase (*srm*) knockdown was performed using siRNA designed and synthesized by GenePharma. The sequences of negative control (scrambled *si*RNA) and *si*RNA targeting spermidine synthase of zebrafish are listed in Table S6. When the concentration of ZFL cells reached more than 80%, the medium was changed and 0.3 μmol/L scrambled *si*RNA or srm-targeting *si*RNA was transfected with Lipofectamine RNAi-MAX Transfection Reagent (Invitrogen, 13778930). The knockdown efficiency was determined 24 h after transfection.

In addition, when the concentration of ZFL cells reached more than 80%, the cell medium was supplemented with 2 mmol/L 2-mercaptoethylamine (Coolaber, Beijing, purity 95.0%) or 2 mmol/L cyclohexylamine (Dr. Ehrenstorfer, Germany, purity 98.33%) to competitively inhibit spermidine synthase.

**Morpholino knockdown**

Morpholino oligonucleotides (MO) synthesized by gene Tools (Philomath, OR) were used to knockdown the spermidine synthase gene of zebrafish larvae. The MO sequences are: srm MO, 5’-GCACCGGGAGGACTCACCTTTTAAA-3’, and standard control MO, 5’-CCTCTTACCTCAGTTACAATTTATA-3’. Zebrafish larvae were immersed 50 nM srm MO or standard control MO from 4 dpf.

**Western blotting**

The expression of *srm* in the liver of zebrafish was detected with glyceraldehyde-3-phosphate dehydrogenase (GAPDH) as the internal reference. Zebrafish liver was homogenized in a cold HBSS buffer mixed with 1mM PMSF and phosphatase inhibitors. The content of protein was quantified by the BCA method, which was then scaled to a uniform amount per sample weight. The protein was loaded into a 12% SDS-PAGE for electrophoresis, which was then transferred to a 0.22 μm PVDF membrane (Millipore, USA). The PVDF membrane was treated with primary antibodies targeting anti-GAPDH (CST, 2118, 1:2000) and anti-SRM (Proteintech, 19858-1-AP, 1:1000), followed by the execution of western blotting analysis as previously described [10].

**Statistical analysis**

After analysis using GraphPad Prism 8, all data were expressed as mean ± SEM or median (IQR). All analyses were done using one-way ANOVA followed by Duncan’s multiple range test (more than two groups) and the student’s t-test (two groups). The relative abundance of gut microbiota sequencing was compared by using Kruskal-Wallis test. The statistical test and number of samples used are included and described in each figure legend.

**Figure legends**

**Figure S1 The series of experiments conducted in this study.**

**Figure S2 The effects of diet supplementation of mannose on growth performance and liver health of zebrafish.**

One-month old zebrafish were fed a normal-fat diet (NFD), high-fat diet (HFD) and three treatment diets supplemented with 2.5 g/kg (2.5M), 5 g/kg (5M) and 10 g/kg (10M) mannose for four weeks, respectively. **(A)** Survival rate of zebrafish (n = 5). **(B)** Weight gain rate of zebrafish (n = 5). **(C)** Feed conversion ratio of zebrafish (n = 5). **(D)** The expression of liver lipogenesis genes (n = 4-5). **(E)** The expression of liver lipolysis genes (n = 4-5). **(F)** The gene expression of liver pro-inflammatory factors (n = 4-5). **(G)** The gene expression of liver anti-inflammatory factors (n = 4-5). Data were presented as mean ± SEM. One-way ANOVA was employed to compare the groups, followed by Duncan’s multiple range test. Distinct letters denote significant differences at (*p* < 0.05).

**Figure S3 The effects of diet supplementation of mannose on the gut health of zebrafish.**

**(A)** Gut H&E sections (n = 3). **(B)** Gut injury scores (n = 3). **(C)** Lipopolysaccharides (LPS) content in zebrafish serum (n = 4). **(D)** The expression of intestinal barrier-related genes (n = 4-5). **(E)** The gene expression of intestinal pro-inflammatory factors (n = 4-5). **(F)** The gene expression of intestinal anti-inflammatory factors (n = 4-5). Data were presented as mean ± SEM. One-way ANOVA was employed to compare the groups, followed by Duncan’s multiple range test. Distinct letters denote significant differences at (*p* < 0.05).

**Figure S4 The effects of diets supplementation of different levels of mannose on** **gut microbiota of zebrafish.**

**(A)** The relative abundance of gut microbiota at the phylum level (n = 5). **(B)** The relative abundance of gut microbiota at the genus level (n = 5). **(C)** Alpha diversity analysis (Chao1 and Shannon) of gut microbiota (n = 5). **(D)** LEfse analysis of gut microbiota at the species level (n = 5). **(E)** PCoA analysis (Bray-Curtis) of gut microbiota at the phylum level (n = 5). **(F)** PCoA analysis (Bray-Curtis) of gut microbiota at the genus level (n = 5). Data were presented as mean ± SEM.

**Figure S5** **Meta analysis fish gut microbiota.**

**(A)** The relationship between *Plesiomonas* and the liver TAG content in zebrafish by Spearman correlation analysis. **(B)** The relationship between *Aeromonas* and the liver TAG content in zebrafish by Spearman correlation analysis. **(C)** The relationship between *Cetobacterium* and the liver TAG content in *Cyprinus carpio* by Spearman correlation analysis. **(D)** The relationship between *Plesiomonas* and the liver TAG content in *Cyprinus carpio* by Spearman correlation analysis. **(E)** The relationship between *Aeromonas* and the liver TAG content in *Cyprinus carpio* by Spearman correlation analysis.

**Figure S6** **Genomic analysis representative strains of fish gut.**

**(A)** Genomic sequences of three representative strains of *C. somerae* XMX-1, *Plesiomonas* *shigelloides* (*P. Shigeloides*) CB5 and *Aeromonas veronii* (*A. veronii*) XMX-5. Schematic diagram was created with BioRender.com. **(D)** Venn diagram of all genes of the three representative strains. **(B)** The plasmid circos map, **(E)** KEGG and **(H)** COG functional gene annotations and **(K)** CAZymes of *C. someare* XMX-1, with large number of genes related to carbohydrate metabolism (206 genes) and carbohydrate transport and metabolism (285 genes) with 41 genes involved in glycosyl Transferases as part of CAZymes. **(C)** The plasmid circos map, **(F)** KEGG and **(I)** COG functional gene annotations and **(K)** CAZymes of *P. shigeloides* CB5, with a larger number of genes related to carbohydrate metabolism (175 genes) and carbohydrate transport and metabolism (178 genes), and glycoside hydrolases (31 genes) being the most common among CAZymes. **(G)** KEGG and **(J)** COG functional gene annotations and **(K)** CAZymes of *A. veronii* XMX-5, with a large number of genes related to carbohydrate metabolism (224 genes) and carbohydrate transport and metabolism (208 genes), and glycoside hydrolases (43 genes) being the most common among CAZymes. The growth of **(L)** *C. somerae* XMX-1, **(M)** *P. shigeloides* CB5 and **(N)** *A. veronii* XMX-5 under different carbon sources (GAM medium without obvious carbon source and supplemented with mannose, respectively) (n = 6).

**Figure S7** **The effect of mannose supplementation** **on zebrafish liver steatosis.**

**(A)** Germ-free zebrafish larvae were fed sterile HFD supplemented with 5.0 g/kg mannose for 7 days. Two groups were divided into HFD and Mannose groups. Each group had 5 replicate bottles with 30 zebrafish larvae in each, for a total of 300 zebrafish larvae used. Schematic diagram was created with BioRender.com. **(B)** TAG content of GF zebrafish larvae (n = 5). **(C)** Oil red staining of GF zebrafish larvae (n = 6). **(D)** Quantification of oil red staining of GF zebrafish larvae (n = 6). **(E)** The expression of lipogenesis genes in GF zebrafish larvae (n = 5-6). **(F)** The expression of lipolysis genes in GF zebrafish larvae (n = 5-6). **(G)** The expression of pro-inflammatory factor genes in GF zebrafish larvae (n = 4-6). **(H)** The expression of anti-inflammatory factor genes in GF zebrafish larvae (n = 4-6). Data were presented as mean ± SEM. The student’s t-test was employed to compare between two groups. Non-significant differences were marked as ns.

**Figure S8** **The effect of supplementation of mannose induced gut microbiota on zebrafish liver steatosis.**

**(A)** Germ-free zebrafish larvae that were transferred the gut microbiota from the HFD and Mannose groups were fed a high-fat diet for 7 days. Three groups were divided into GF, HFD-GM and HFD-MO-GM groups. Each group had 5 replicate bottles with 30 zebrafish larvae in each, for a total of 450 zebrafish larvae used. Schematic diagram was created with BioRender.com. **(B)** TAG content of GF zebrafish larvae (n = 5). **(C)** Oil red staining of GF zebrafish larvae (n = 6-7). **(D)** Quantification of oil red staining of GF zebrafish larvae (n = 6-7). **(E)** The expression of lipogenesis genes in GF zebrafish larvae (n = 4-6). **(F)** The expression of lipolysis genes in GF zebrafish larvae (n = 4-6). **(G)** The expression of pro-inflammatory factor genes in GF zebrafish larvae (n = 4-6). **(H)** The expression of anti-inflammatory factor genes in GF zebrafish larvae (n = 4-6). Data were presented as mean ± SEM. The student’s t-test was employed to compare between two groups. Significant differences were marked as **p* < 0.05; ***P* < 0.01, ****p* < 0.001.

**Figure S9 The effect of *C. somerae* supplementation on zebrafish liver steatosis.**

Germ-free zebrafish larvae were fed a high-fat diet and supplemented with 10^6^ CFUs/g *C. somerae* for 7 days. **(A)** The expression of lipogenesis genes in GF zebrafish larvae (n = 5-6). **(B)** The expression of lipolysis genes in GF zebrafish larvae (n = 4-6). **(C)** The expression of pro-inflammatory factor genes in GF zebrafish larvae (n = 4-6). **(D)** The expression of anti-inflammatory factor genes in GF zebrafish larvae (n = 4-6). Data were presented as mean ± SEM. The student’s t-test was employed to compare between two groups. Significant differences were marked as **p* < 0.05; ***P* < 0.01, ****p* < 0.001, *****P* < 0.0001.

**Figure S10** **The effect of supplementation of *P. Shigeloides* and *A. veronii* on zebrafish liver steatosis.**

**(A)** Germ-free zebrafish larvae were directly fed high-fat diet and supplemented with 10^6^ CFUs/g *P. Shigeloides* and *A. veronii* for 7 days. Three different treatment groups (HFD, P. Shigeloides and A. veronii) were used. Each group had 5 replicate bottles with 30 zebrafish larvae in each, for a total of 450 zebrafish larvae used. Schematic diagram was created with BioRender.com. **(B)** TAG content of GF zebrafish larvae (n = 5). **(O)** Oil red staining of GF zebrafish larvae (n = 7). **(P)** Quantification of oil red staining of GF zebrafish larvae (n = 7). Data were presented as mean ± SEM. The student’s t-test was employed to compare between two groups. Significant differences were marked as ns=not significant.

**Figure S11 The effect of *C. somerae* supplementation on zebrafish liver steatosis.**

**(A)** One-month old zebrafish were fed high-fat diets supplemented with 10^6^ CFUs/g *C. somerae* for 4 weeks. Two different treatment groups (HFD and C. somerae) were used. Each group had 5 replicate tanks with 24 zebrafish in each, for a total of 240 zebrafish used. Schematic diagram was created with BioRender.com. **(B)** TAG content in zebrafish liver (n = 5-6). **(C)** Liver H&E section (n = 3). **(D)** Liver injury score (n = 3). **(E)** The expression of liver lipogenesis genes (n = 4-6). **(F)** The expression of liver lipolysis genes (n = 6). **(G)** The gene expression of liver pro-inflammatory factors (n = 5-6). **(H)** The gene expression of liver anti-inflammatory factors (n = 4-6). **(I)** ALT, **(J)** AST and **(K)** LPS content in zebrafish serum (n = 4). **(L)** Gut H&E section (n = 3). **(M)** Gut injury score (n = 3). Data were presented as mean ± SEM. The student’s t-test was employed to compare between two groups. Significant differences were marked as **p* < 0.05; ***P* < 0.01, ****p* < 0.001.

**Figure S12** **Metabolite analysis of *C. somerae* XMX-1.**

**(A)** Correlation analysis of quality control of samples in the full spectrum metabolome (n = 4). **(B)** CV distribution diagram of each group of samples in the full spectrum metabolome (n = 4). **(C)** The sample hierarchical clustering tree (n = 4). **(D)** Venn diagram of differential metabolites (n = 4). **(E)** Clustering heat map of differential metabolites (n = 4). **(F)** Bar chart of differential metabolites (n = 4). **(G)** The contents of metabolites ^13^C_4_^15^N_2_-putrescine, ^13^C_4_^15^N_2_-spermidine, putrescine and spermidine in bacterial pellet of *C. somerae* XMX-1 were detected after adding ^13^C_6_^15^N_4_-arginine dihydrochloride (final concentration 680 μM) to GAM medium for 24h (n = 4). **(H)** The contents of metabolites D_8_-putrescine, D_8_-spermidine, putrescine and spermidine in bacterial pellet of *C. somerae* XMX-1 were detected after adding D_8_-putrescine dihydrochloride (final concentration 250 μM) to GAM medium for 24h (n = 4). Data were presented as mean ± SEM.

**Figure S13** **The metabolism of putrescine to spermidine exerts the effect of lowering liver fat in** **ZFL cells.**

**(A)** The downstream metabolite D_8_-spermidine was detected after D_8_-putrescine dihydrochloride (final concentration 200 μM) was supplemented in ZFL cell medium for 24h. **(B)** ZFL cells were treated with 100μmol/L oleic acid to construct the lipid accumulation model, and the treatment group was supplemented with 200 μM putrescine and 200 μM spermidine for 24h, respectively. **(C)** TAG content of ZFL cells (n = 6). **(D)** The expression of lipogenesis genes in ZFL cells induced by putrescine and spermidine (n = 5-6). **(E)** The expression of lipolysis genes in ZFL cells induced by putrescine and spermidine (n = 5-6). **(F)** The expression of pro-inflammatory genes in ZFL cells induced by putrescine and spermidine (n = 5-6). **(G)** The expression of anti-inflammatory genes in ZFL cells induced by putrescine and spermidine (n = 5-6). **(H)** The expression of *srm* gene in ZFL cells induced by putrescine and spermidine (n = 6). **(I)** The lipid-lowering effect evaluation of 200 μM putrescine and 200 μM spermidine on ZFL cells after 75 pmol siRNA 632 knockdown of spermidine synthase, respectively. **(J)** The lipid-lowering effect of 200 μM putrescine and 200 μM spermidine on ZFL cells after competitive inhibition of spermidine synthase by 2 mmol/L 2-mercaptoethylamine and 2 mmol/L cyclohexylamine, respectively. **(K)** TAG content of ZFL cells after competitive inhibition of spermidine synthase by 2-mercaptoethylamine (n = 5-6). **(L)** TAG content of ZFL cells after competitive inhibition of spermidine synthase by cyclohexylamine. Data were presented as mean ± SEM. The student’s t-test was employed to compare between two groups. Significant differences were marked as **p* < 0.05, ***p* < 0.01, ****P* < 0.001, *****p* < 0.0001, ns=not significant. **(A)**, **(B)**, **(I)** and **(J)** were created with BioRender.com.

**Figure S14** **The metabolism of putrescine to spermidine exerts the effect of lowering liver fat in** **zebrafish larvae.**

**(A)** The lipid-lowering effect evaluation of 0.5 g/kg putrescine and 0.8 g/kg spermidine on zebrafish larvae after 50 nM *vivo* MO srm-201 knockdown of spermidine synthase. Each group had 6 replicate bottles with 30 zebrafish larvae in each, for a total of 1080 zebrafish larvae used. Schematic diagram was created with BioRender.com. **(B)** Oil red staining of zebrafish larvae (n = 6). **(C)** Quantification of oil red staining of zebrafish larvae (n = 6) after 50 nM *vivo* MO srm-201 knockdown of spermidine synthase. Data were presented as mean ± SEM. The student’s t-test was employed to compare between two groups. Significant differences were marked as ***p* < 0.01, *****p* < 0.0001, ns=not significant.

**Figure S15 The mechanism of putrescine and spermidine on reducing liver fat in zebrafish.**

**(A)** One-month old zebrafish were fed with diets supplemented with 0.5 g/kg putrescine and 0.8 g/kg spermidine (with equimolar amounts of putrescine and spermidine) for four weeks. Four groups were divided into normal-fat diet (NFD), high-fat diet (HFD), 0.05putrescine and 0.08spermidine groups. Each group had 5 replicate tanks with 24 zebrafish in each, for a total of 480 zebrafish used. Schematic diagram was created with BioRender.com. **(B)** Oil red staining of zebrafish liver (n = 3). The expression of **(C)** lipogenesis, **(D)** lipolysis, **(E)** pro-inflammatory factors and **(F)** anti-inflammatory factor genes was detected (n = 5-6) on the zebrafish fatty liver model. **(G)** The content of putrescine in liver (n = 5-6). **(H)** The content of spermidine in liver (n = 5-6). **(I)** Linear regression analysis of the content of putrescine and spermidine in liver (n = 25). **(J)** Polyamine metabolites (putrescine, spermidine and spermine) were analyzed in both the normal-fat diet and high-fat diet. **(K)** Polyamine metabolites (putrescine, spermidine and spermine) were analyzed in both the normal-fat diet and high-fat diet. **(L)** Western blot analysis of spermidine synthase in liver (n = 3). **(M)** Quantification of the expression of liver spermidine synthase, using GAPDH as a control (n = 3). **(N)** The expression of liver polyamine metabolism related genes (n = 5-6). Data were presented as mean ± SEM. The student’s t-test was employed to compare between two groups. Significant differences were marked as **p* < 0.05, ***p* < 0.01, ****p* < 0.001.

**Figure S16 The polyamine metabolism in zebrafish.**

**(A)** LC-MS targeted quantitative analysis was used to measure the levels of Arginine, Putrescine and Spermidine in the gut contents of zebrafish (n = 3). Germ-free zebrafish larvae were transferred with 10^6^ CFUs/g *C. somerae* after 50 nM *vivo* MO srm-201 knockdown of spermidine synthase. Each group had 4 replicate bottles with 30 zebrafish larvae in each, for a total of 480 zebrafish larvae used. **(B)** The content of putrescine in zebrafish larvae (n = 4). **(C)** The content of spermidine in zebrafish larvae (n = 4). Data were presented as mean ± SEM. The student’s t-test was employed to compare between two groups. Significant differences were marked as **p* < 0.05, ***p* < 0.01, ****p* < 0.001, ns=not significant.

**Table S1.** Ingredients (g/100g diet) and chemical compositions (%) of zebrafish diets

| Ingredients (g/100g diet) | NFD | HFD | 2.5M | 5M | 10M | *C. somerae* | 0.05Putrescine | 0.08Spermidine |
| --- | --- | --- | --- | --- | --- | --- | --- | --- |
| Casein | 40.00 | 40.00 | 40.00 | 40.00 | 40.00 | 40.00 | 40.00 | 40.00 |
| Gelatin | 10.00 | 10.00 | 10.00 | 10.00 | 10.00 | 10.00 | 10.00 | 10.00 |
| Dextrin | 28.00 | 16.00 | 15.75 | 15.50 | 15.00 | 16.00 | 16.00 | 16.00 |
| Lard oil | 3.00 | 8.00 | 8.00 | 8.00 | 8.00 | 8.00 | 8.00 | 8.00 |
| Soybean oil | 3.00 | 8.00 | 8.00 | 8.00 | 8.00 | 8.00 | 8.00 | 8.00 |
| Lysine | 0.33 | 0.33 | 0.33 | 0.33 | 0.33 | 0.33 | 0.33 | 0.33 |
| VC phosphate | 0.10 | 0.10 | 0.10 | 0.10 | 0.10 | 0.10 | 0.10 | 0.10 |
| Vitamin premix^1^ | 0.40 | 0.40 | 0.40 | 0.40 | 0.40 | 0.40 | 0.40 | 0.40 |
| Mineral premix^2^ | 0.40 | 0.40 | 0.40 | 0.40 | 0.40 | 0.40 | 0.40 | 0.40 |
| Monocalcium phosphate | 2.00 | 2.00 | 2.00 | 2.00 | 2.00 | 2.00 | 2.00 | 2.00 |
| Choline chloride | 0.20 | 0.20 | 0.20 | 0.20 | 0.20 | 0.20 | 0.20 | 0.20 |
| Sodium alginate | 2.00 | 2.00 | 2.00 | 2.00 | 2.00 | 2.00 | 2.00 | 2.00 |
| Microcrystalline cellulose | 4.00 | 4.00 | 4.00 | 4.00 | 4.00 | 4.00 | 4.00 | 4.00 |
| Zeolite | 6.57 | 8.57 | 8.57 | 8.57 | 8.57 | 8.57 | 8.52 | 8.49 |
| Mannose^3^ | 0 | 0 | 0.25 | 0.50 | 1.00 | 0 | 0 | 0 |
| *C. somerae*^4^ | 0 | 0 | 0 | 0 | 0 | 10^6^ CFUs/g | 0 | 0 |
| Putrescine^5^ | 0 | 0 | 0 | 0 | 0 | 0 | 0.05 | 0 |
| Spermidine^6^ | 0 | 0 | 0 | 0 | 0 | 0 | 0 | 0.08 |
| Total | 100.00 | 100.00 | 100.00 | 100.00 | 100.00 | 100.00 | 100.00 | 100.00 |
| Proximate analysis |  |  |  |  |  |  |  |  |
| Crude protein (%) | 42.19 | 42.19 | 42.19 | 42.19 | 42.19 | 42.19 | 42.19 | 42.19 |
| Crude fat (%) | 6.00 | 16.00 | 16.00 | 16.00 | 16.00 | 16.00 | 16.00 | 16.00 |

1. Containing the following (g/kg vitamin premix): thiamine, 0.438; riboflavin, 0.632; pyridoxine⋅HCl, 0.908; d-pantothenic acid, 1.724; nicotinic acid, 4.583; biotin, 0.211; folic acid, 0.549; vitamin B-12, 0.001; inositol, 21.053; menadione sodium bisulfite, 0.889; retinyl acetate, 0.677; cholecalciferol, 0.116; dl-α-tocopherol-acetate,12.632.

2. Containing the following (g/kg mineral premix): CoCl_2_⋅6H_2_O, 0.074; CuSO_4_⋅5H_2_O, 2.5; FeSO_4_⋅7H_2_O, 73.2; NaCl, 40.0; MgSO_4_⋅7H_2_O, 284.0; MnSO_4_⋅H_2_O, 6.50; KI, 0.68; Na_2_SeO_3_, 0.10; ZnSO_4_⋅7H_2_O, 131.93; Cellulose, 501.09.

3. Mannose was purchased from Yuanye, Shanghai, China.

4. *Cetobacterium somerae* (*C. somerae*)

5. Putrescine was purchased from Anpel, Shanghai, China.

6. Spermidine was purchased from Anpel, Shanghai, China.

**Table S2.** Ingredients (g/100g diet) and chemical compositions (%) of zebrafish larvae diets

| Ingredients (g/100g diet) | HFD | Mannose | 0.05Putrescine | 0.08Spermidine |
| --- | --- | --- | --- | --- |
| Casein | 46.00 | 46.00 | 46.00 | 46.00 |
| Gelatin | 11.00 | 11.00 | 11.00 | 11.00 |
| Dextrin | 10.00 | 9.50 | 10.00 | 10.00 |
| Lard oil | 8.00 | 8.00 | 8.00 | 8.00 |
| Soybean oil | 8.00 | 8.00 | 8.00 | 8.00 |
| Fish liver oil | 4.00 | 4.00 | 4.00 | 4.00 |
| Soybean lecithin | 2.00 | 2.00 | 2.00 | 2.00 |
| Lysine | 0.37 | 0.37 | 0.37 | 0.37 |
| VC phosphate | 0.10 | 0.10 | 0.10 | 0.10 |
| Vitamin premix^1^ | 0.40 | 0.40 | 0.40 | 0.40 |
| Mineral premix^2^ | 0.40 | 0.40 | 0.40 | 0.40 |
| Monocalcium phosphate | 2.00 | 2.00 | 2.00 | 2.00 |
| Choline chloride | 0.20 | 0.20 | 0.20 | 0.20 |
| Sodium alginate | 4.00 | 4.00 | 4.00 | 4.00 |
| Zeolite | 3.53 | 3.53 | 3.48 | 3.45 |
| Mannose^3^ | 0 | 0.50 | 0 | 0 |
| Putrescine^4^ | 0 | 0 | 0.05 | 0 |
| Spermidine^5^ | 0 | 0 | 0 | 0.08 |
| Total | 100.00 | 100.00 | 100.00 | 100.00 |
| Proximate analysis |  |  |  |  |
| Crude protein (%) | 48.09 | 48.09 | 48.09 | 48.09 |
| Crude fat (%) | 22.00 | 22.00 | 22.00 | 22.00 |

1. Containing the following (g/kg vitamin premix): thiamine, 0.438; riboflavin, 0.632; pyridoxine⋅HCl, 0.908; d-pantothenic acid, 1.724; nicotinic acid, 4.583; biotin, 0.211; folic acid, 0.549; vitamin B-12, 0.001; inositol, 21.053; menadione sodium bisulfite, 0.889; retinyl acetate, 0.677; cholecalciferol, 0.116; dl-α-tocopherol-acetate,12.632.

2. Containing the following (g/kg mineral premix): CoCl_2_⋅6H_2_O, 0.074; CuSO_4_⋅5H_2_O, 2.5; FeSO_4_⋅7H_2_O, 73.2; NaCl, 40.0; MgSO_4_⋅7H_2_O, 284.0; MnSO_4_⋅H_2_O, 6.50; KI, 0.68; Na_2_SeO_3_, 0.10; ZnSO_4_⋅7H_2_O, 131.93; Cellulose, 501.09.

3. Mannose was purchased from Yuanye, Shanghai, China.

4. Putrescine was purchased from Anpel, Shanghai, China.

5. Spermidine was purchased from Anpel, Shanghai, China.

**Table S3.** A real-time PCR amplification system

| Ingredient | Volume |
| --- | --- |
| cDNA | 2 μL |
| Forward primer（10 μM） | 0.5 μL |
| Reverse primer（10 μM） | 0.5 μL |
| 2×SYBR® GreenSupermix | 5 μL |
| RNase-Free ddH_2_O | 2 μL |
| Total | 10 μL |

**Table S4.** A real-time PCR amplification program

| Program Name | Cycles | Target (℃) | Time (mm:ss) |
| --- | --- | --- | --- |
| Preheat | 1 | 95 | 02:00 |
| Quantification | 40 | 95 | 00:05 |
|  |  | 60 | 00:10 |
|  |  | 72 | 00:20 |
| Melting Curves | 1 | 95 | 00:05 |
|  |  | 60 | 00:05 |
|  |  | 95 |  |
| End | 1 | 37 | 00:05 |

**Table S5.** List of primer sequences used for qPCR

| Genes | Full name of the genes and its function | Forward primer (5′ –3′) | Reverse primer (5′ –3′) | Reference |
| --- | --- | --- | --- | --- |
| *rps11* | *Ribosomal protein s11* Internal reference gene | ACAGAAATGCCCCTTCACTG | GCCTCTTCTCAAAACGGTTG | [8] |
| *srebf1* | *Sterol Regulatory Element Binding Transcription Factor 1* Lipogenesis gene | CAGAGGGTGGGCATGCTGGC | CAGAGGGTGGGCATGCTGGC | [7] |
| *c/ebpα* | *CCAAT Enhancer Binding Protein Alpha* Lipogenesis gene | AACGGAGCGAGCTTGACTT | AAATCATGCCCATTAGCTGC | [8] |
| *pparγ* | *Peroxisome proliferator-activated receptor Gamma* Lipogenesis gene | CCTGTCCGGGAAGACCAGCG | GTGCTCGTGGAGCGGCATGT | [8] |
| *acc* | *Acetyl-CoA Carboxylase* Lipogenesis gene | GCGTGGCCGAACAATGGCAG | GCAGGTCCAGCTTCCCTGCG | [8] |
| *fas* | *Fatty Acid Synthase* Lipogenesis gene | GGAGCAGGCTGCCTCTGTGC | TTGCGGCCTGTCCCACTCCT | [8] |
| *dgat2* | *Diacylglycerol O-Acyltransferase 2*  Lipogenesis gene | CCATACTTGCTGCATATTCC | ATGTCATGATAAACTGCAGC | [8] |
| *pparα* | *Peroxisome Proliferator Activated Receptor Alpha* Lipolysis gene | CTGCGGGACATCTCTCAGTC | ACCGTAAACACCTGACGACG | [8] |
| *acox3* | *Acyl-CoA Oxidase 3* Lipolysis gene | TGGAAGGACATGATGCGCTTT | AGGCTGCCGGGCAAAAA | [7] |
| *cpt1* | *Carnitine Palmitoyltransferase 1* Lipolysis gene | GCATTGACCTTCAGCTCAGC | CTGCCAACACCAGCACGAAC | [8] |
| *nf-κb p65* | *Nuclear Factor Kappa-B P65* Pro-inflammatory factor | CCGTCGTCTCTCAGCCGAT | AGAAGCGCACCTCAATGTCC | [11] |
| *tnf-α* | *Tumor Necrosis Factor Alpha* Pro-inflammatory factor | GCGCTTTTCTGAATCCTACG | TGCCCAGTCTGTCTCCTTCT | [11] |
| *il-1β* | *Interleukin 1 Beta* Pro-inflammatory factor | TGGACTTCGCAGCACAAAATG | GTTCACTTCACGCTCTTGGATG | [11] |
| *il-6* | *Interleukin 6* Pro-inflammatory factor | TCAACTTCTCCAGCGTGATG | TCTTTCCCTCTTTTCCTCCTG | [8] |
| *il-10* | *Interleukin 10* Anti-inflammatory factor | TCACGTCATGAACGAGATCC | CCTCTTGCATTTCACCATATCC | [8] |
| *tgf-β* | *Transforming Growth Factor Beta* Anti-inflammatory factor | CAAACCAGCAGAGCACGGATA | CGGTGCCAACAGCTCGTCTC | [11] |
| *claudin 7* | *Claudin 7* Intestinal barrier-related gene | CTTGCTCAAAGGGTCAGTCA | GTCCTTTCCAGCTCGTGAAC | [8] |
| *occludin b* | *Occludin b*  Intestinal barrier-related gene | CAAAATCAGGCAAAGGCTTC | AACAATAGTGGCGATGAGCA | [8] |
| *arg2* | *Arginase2* Polyamine metabolism | GGCCAGTCTGTAGCTTTCTTACTAAA | ATCTCTAAGACCAATGTACACCAGATC | This study |
| *odc1* | *Ornithine decarboxylase 1* Polyamine metabolism | CATCTCAGATGCTCGCTATGTTTTC | GTATTTATCCAGCGCAGGGTTAATAAC | This study |
| *srm* | *Spermidine Synthase* Polyamine metabolism | TTCAGTGTGAGATTGATGAGGATGT | TCTTCATGAATTCAAAGCCATCACC | This study |
| *sms* | *Spermine synthase* Polyamine metabolism | TTCAAGAGCAGGAAATGACAGAGAATG | TTTCTCCAGTGCATTCAGTAGATTGTC | This study |

**Table S6.** Sequences of *si*RNA.

| *si*RNA | Forward primer (5′ –3′) | Reverse primer (5′ –3′) |
| --- | --- | --- |
| Scrambled *si*RNA | UUCUCCGAACGUGUCACGUTT | ACGUGACACGUUCGGAGAATT |
| srm-632 | CCGGCUGAAAGCUUGUUCATT | UGAACAAGCUUUCAGCCGGTT |

**REFERENCES**

1.	Matsumoto, Mitsuharu, Yoshimi Benno. 2013. The relationship between microbiota and polyamine concentration in the human intestine: A pilot study. *Microbiology and Immunology* 51(1): 25-35. <https://doi.org/10.1111/j.1348-0421.2007.tb03887.x>

2.	Núñez‐Sánchez, María Ángeles, María Antonia Martínez‐Sánchez, Marta Sierra‐Cruz, Ana Lambertos, Sara Rico‐Chazarra, Alba Oliva‐Bolarín, Andrés Balaguer‐Román, José Enrique Yuste, Carlos Manuel Martínez, Adriana Mika, María Dolores Frutos, Camilo J. Llamoza‐Torres, José Córdoba‐Chacón, Bruno Ramos‐Molina. 2024. Increased hepatic putrescine levels as a new potential factor related to the progression of metabolic dysfunction‐associated steatotic liver disease. *Journal of Pathology* 264(1): 101-11. <https://doi.org/10.1002/path.6330>

3.	Ma, Yanfei, Yifan Zhong, Wenjie Tang, Teresa G. Valencak, Jingliang Liu, Zhaoxi Deng, Jiangdi Mao, Daren Liu, Shanshan Wang, Yuhao Wang, Haifeng Wang. 2025. *Lactobacillus reuteri* zj617 attenuates metabolic syndrome via microbiota-derived spermidine. *Nature Communications* 16(1): 877. <https://doi.org/10.1038/s41467-025-56105-4>

4.	Wang, Tixiao, Yuchen Fan, Siyu Tan, Zehua Wang, Mengzhen Li, Xiaowei Guo, Xiangguo Yu, Qinghai Lin, Xiaojia Song, Leiqi Xu, Lixiang Li, Shiyang Li, Lifen Gao, Xiaohong Liang, Chunyang Li, Chunhong Ma. 2024. Probiotics and their metabolite spermidine enhance ifn-γ+cd4+ t cell immunity to inhibit hepatitis b virus. *Cell Reports Medicine* 5(11): 101822. <https://doi.org/10.1016/j.xcrm.2024.101822>

5.	Rawls, John F. , Buck S. Samuel, Jeffrey I. Gordon. 2004. Gnotobiotic zebrafish reveal evolutionarily conserved responses to the gut microbiota. *Proceedings of the National Academy of Sciences of the United States of America* 101(13): 4596-601. <https://doi.org/10.1073/pnas.0400706101>

6.	Pham, Linh N., Michelle Kanther, Ivana Semova, John F. Rawls. 2008. Methods for generating and colonizing gnotobiotic zebrafish. *Nature Protocols* 3(12): 1862-75. <https://doi.org/10.1038/nprot.2008.186>

7.	Ran, Chao, Mingxu Xie, Jie Li, Yadong Xie, Qianwen Ding, Yu Li, Wei Zhou, Yalin Yang, Zhen Zhang, Rolf Erik Olsen, Zhigang Zhou. 2021. Dietary nucleotides alleviate hepatic lipid deposition via exogenous amp-mediated ampk activation in zebrafish. *Journal of Nutrition* 151(10): 2986-96. <https://doi.org/10.1093/jn/nxab232>

8.	Zhang, Zhen, Chao Ran, Qian-wen Ding, Hong-liang Liu, Ming-xu Xie, Ya-lin Yang, Ya-dong Xie, Chen-chen Gao, Hong-ling Zhang, Zhi-gang Zhou. 2019. Ability of prebiotic polysaccharides to activate a hif1α-antimicrobial peptide axis determines liver injury risk in zebrafish. *Communications Biology* 2(1): 274. <https://doi.org/10.1038/s42003-019-0526-z>

9.	Wang, Anran, Zhen Zhang, Qianwen Ding, Yalin Yang, Jérôme Bindelle, Chao Ran, Zhigang Zhou. 2021. Intestinal *cetobacterium* and acetate modify glucose homeostasis via parasympathetic activation in zebrafish. *Gut Microbes* 13(1): 1-15. <https://doi.org/10.1080/19490976.2021.1900996>

10.	Zhang, Zhen, Hong‐Ling Zhang, Da‐Hai Yang, Qiang Hao, Hong‐Wei Yang, De‐Long Meng, Willem Meindert de Vos, Le‐Luo Guan, Shu‐Bin Liu, Tsegay Teame, Chen‐Chen Gao, Chao Ran, Ya‐Lin Yang, Yuan‐Yuan Yao, Qian‐Wen Ding, Zhi‐Gang Zhou. 2024. *Lactobacillus rhamnosus* gg triggers intestinal epithelium injury in zebrafish revealing host dependent beneficial effects. *iMeta* 3(2): e181. <https://doi.org/10.1002/imt2.181>

11.	Li, Shenghui, Hongwei Yang, Ya Jin, Qiang Hao, Shubin Liu, Qianwen Ding, Yuanyuan Yao, Yalin Yang, Chao Ran, Chenglong Wu, Shengkang Li, Kaimin Cheng, Jun Hu, Hongliang Liu, Zhen Zhang, Zhigang Zhou. 2023. Dietary cultured supernatant mixture of *cetobacterium somerae* and *lactococcus lactis* improved liver and gut health, and gut microbiota homeostasis of zebrafish fed with high-fat diet. *Fish & Shellfish Immunology* 142: 109139. <https://doi.org/10.1016/j.fsi.2023.109139>
